# Anticipatory capture of circulating peptidergic vesicles in a clock neuron

**DOI:** 10.1091/mbc.E25-11-0558

**Published:** 2026-04-06

**Authors:** Markus K. Klose, Junghun Kim, Brigitte F. Schmidt, David L. Deitcher, Edwin S. Levitan

**Affiliations:** ^a^Department of Pharmacology and Chemical Biology, University of Pittsburgh, Pittsburgh, PA 15261; ^b^Department of Chemistry, Carnegie Mellon University, Pittsburgh, PA 15213; ^c^Department of Neurobiology and Behavior, Cornell University, Ithaca, NY 14853; Brandeis University

## Abstract

Neuropeptide release by *Drosophila* sLNv clock neurons controls circadian behaviors and sleep. Strikingly, neuropeptide content in sLNv terminals is rhythmic with late-night accumulation occurring, while the axon arbor is expanding in preparation for midmorning synaptic exocytosis of neuropeptide-containing dense-core vesicles (DCV). Past studies showed that increased synaptic neuropeptide content can be produced by delivery of more neuropeptide to terminals or activity-dependent capture of circulating DCVs. To distinguish between these mechanisms, neuropeptide-containing DCVs were imaged in the *ex vivo* brain explant preparation. First, postexocytosis DCV axonal transport and presynaptic neuropeptide accumulation following retrograde transport inhibition show that sLNv DCVs circulate. Furthermore, anterograde transport to terminals is constant throughout the day demonstrating there is no increase in DCV delivery. Rather, capture of circulating DCVs produces the daily boost in terminal neuropeptide content. Remarkably, this capture occurs before the daily increase in Ca^2+^ spike activity and is independent of concurrent IP_3_ signaling and axon arbor expansion. Finally, a *per* clock gene mutation inhibits rhythmic DCV capture. Thus, rather than responding to Ca^2+^ signaling or axonal plasticity, capture of circulating DCVs in sLNv presynapses is increased by the molecular clock in anticipation of activity-induced release hours later.

## Abstract (executive summary)

- Clock neurons release neuropeptides packaged in dense-core vesicles (DCV) to control rhythmic behaviors. In *Drosophila* sLNv neurons, synaptic neuropeptide levels increase daily by an undefined posttranscriptional mechanism to promote midmorning release.
- Imaging of sLNv axonal DCV transport in the living brain shows that the increase in synaptic neuropeptide levels is produced by late-night capture of circulating DCVs. Furthermore, this capture is controlled by the molecular clock without the involvement of concurrent axonal plasticity and Ca^2^^+^ signaling.
- Rather than responding to activity or morphological plasticity, clock-dependent vesicle capture at presynapses occurs in anticipation of the peptidergic transmission required for controlling rhythmic behaviors.

## INTRODUCTION

*Drosophila* sLNv clock neurons release two neuropeptides (PDF and sNPF) that are copackaged in the same dense-core vesicles (DCV) (Yu *et al.*, 2025) to regulate circadian behavior (Crespo-Flores and Barber, 2022) and sleep (Shang *et al.*, 2013). Interestingly, PDF content in sLNv neurons is rhythmic based on the function of the circadian clock (Park *et al.*, 2000), which is a feature shared by neuropeptides in other clock neurons (Hermann-Luibl *et al.*, 2014; Fujiwara *et al.*, 2018; Díaz *et al.*, 2019; Sekiguchi *et al.*, 2024). For both PDF and an exogenously expressed fluorescent protein-tagged neuropeptide, activity-dependent DCV exocytosis decreases presynaptic sLNv terminal neuropeptide content at midmorning (Park *et al.*, 2000; Klose *et al.*, 2021; 2026). Furthermore, this synaptic release is preceded by a late-night posttranscriptional upregulation of terminal neuropeptide content (Park *et al.*, 2000; Klose *et al.*, 2021) that is advantageous because synaptic neuropeptide release scales with presynaptic neuropeptide content (Bulgari *et al.*, 2014). However, the mechanism for the daily increase in terminal neuropeptide content is not known.

Traditionally, it was thought that newly synthesized DCVs are delivered to terminals from the soma for exocytotic release by anterograde axonal transport and eventually delivered back to the soma for degradation by retrograde axonal transport (Alonso and Assenmacher, 1983). In this context, the observed decrease in soma neuropeptide content preceding the increase in terminal neuropeptide content in sLNv terminals (Park *et al.*, 2000; Klose *et al.*, 2021) is consistent with a surge in anterograde transport producing the daily increase in terminal neuropeptide content (Supplemental Figure S1A). However, a recent study showed there is late-night DCV exocytosis at the sLNv soma (Klose *et al.*, 2021), which might fully account for the somatic neuropeptide decrease. In the latter case, another mechanism must explain the daily neuropeptide increase in terminals. Such a mechanism was discovered in the peripheral nervous system, where activity induces capture of DCVs as they circulate between distal terminal boutons and an axonal region near the soma to replenish synaptic neuropeptide content following stimulated neuropeptide release (Shakiryanova *et al.*, 2006; Wong *et al.*, 2012; Cavolo *et al.*, 2016). Experimentally, induced capture is evident from a drop in retrograde DCV transport that accompanies increased synaptic neuropeptide content (Shakiryanova *et al.*, 2006) (Supplemental Figure S1B). Thus, two mechanisms for increasing synaptic neuropeptide content are distinguished by their effects on axonal transport: either retrograde vesicle transport is decreased by synaptic capture of vesicles undergoing circulation, or anterograde transport is increased to upregulate neuropeptide delivery to synapses. Although DCV axonal transport has been imaged in the living brain (e.g., Knabbe *et al.*, 2018; Nassal *et al.*, 2022), the role of these mechanisms for regulating daily changes in neuropeptide content is not known.

Here, axonal transport and presynaptic accumulation of neuropeptide-containing DCVs are imaged in the adult *Drosophila* brain. First, time-lapse imaging following synaptic kiss-and-run exocytosis and genetic inhibition of retrograde transport demonstrates that DCVs in sLNv axons are circulating. Furthermore, rhythmic accumulation of DCVs in sLNv terminals occurs without a change in anterograde axonal transport. Instead, a late-night decrease in retrograde transport reveals an enhancement of vesicle capture that upregulates neuropeptide content in the sLNv terminals. Because DCV capture occurs hours before the daily rise in sLNv neuron activity (Klose *et al.*, 2026), follow-up experiments address whether rhythmic presynaptic DCV capture depends on concurrent IP_3_ signaling (Klose *et al.*, 2021), axon arbor plasticity (Fernández *et al.*, 2008), and the circadian clock.

## RESULTS AND DISCUSSION

### Vesicle circulation delivers neuropeptide to presynaptic boutons in sLNv clock neurons

**FIGURE 1:**
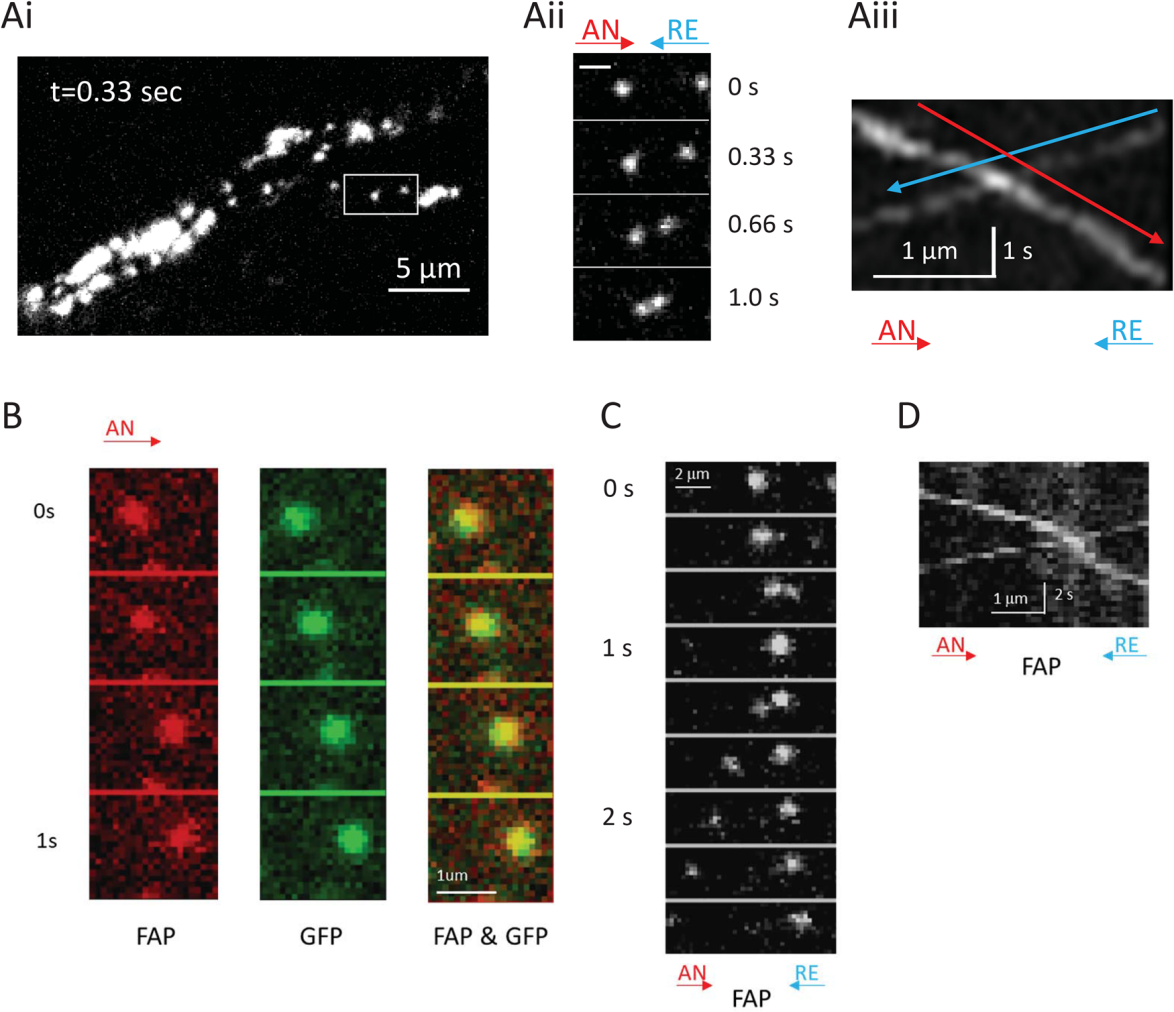
Postexocytosis DCVs display anterograde and retrograde axonal transport. (A) sLNv projections containing DCVs labeled with Dilp2-GFP were imaged at 3 Hz at ZT 3. (Ai) White box reveals the ROI for analysis. (Aii) ROI images at *t* = 0, 0.33, 0.66, and 1.0 s (red arrow indicates anterograde [AN] transport and blue arrow indicates retrograde [RE] transport; scale bar, 1 µm). (Aiii) Kymograph generated from the Ai ROI. (B) Colocalization of GFP and FAP signal in a punctum moving through sLNv nerve terminals of *UAS-Dilp2-GFP, UAS-Dilp2-FAP; PDF-GAL4* flies. (C) Post-exocytosis FAP-labeled puncta in sLNv nerve terminals reveal both anterograde and retrograde transport. (D) Kymograph revealing both anterograde (slope down, left to right) and retrograde (slope down, right to left) axonal transport of postexocytosis DCVs in sLNv projections. Anterograde and retrograde transport of postexocytosis DCVs was seen in multiple neurons of seven brain explants.

To address the basis of the daily upregulation of neuropeptide content in sLNv terminals, we imaged DCV axonal transport in the adult *ex vivo* brain explant preparation from animals with cell-specific expression of the DCV marker Dilp2-GFP (Wong *et al.*, 2012), which recapitulates the known PDF content rhythm in sLNv terminals (Klose *et al.*, 2021). Time-lapse imaging in the morning detected DCVs in distal dorsal sLNv axons (Helfrich-Förster *et al.*, 2007) undergoing both anterograde and retrograde axonal transport (Figure 1A; Supplemental Movie S1). The ability to detect DCV axonal transport in sLNv neurons then led us to formulate experiments for determining the basis of the daily increase in neuropeptide stores.

As capture of circulating DCVs could contribute to the neuropeptide rhythm in sLNv clock neurons, we first sought to test for vesicle circulation in these brain neurons. Vesicle circulation was established by imaging the body wall innervation of the two-dimensional filleted larva and conducting FRAP (fluorescence recovery after photobleaching) experiments with a scanning confocal microscope (Shakiryanova *et al.*, 2006; Wong *et al.*, 2012; Cavolo *et al.*, 2016). These experiments are not feasible in the three-dimensional *ex vivo* brain explant preparation because light scattering limits illumination and collection of fluorescence, and photobleaching illumination produces diffusing radicals that induce extensive photodamage. Therefore, we used an alternative approach based on imaging with the spinning-disk confocal microscope, which is very sensitive and produces relatively little photobleaching. Specifically, experiments took advantage of 1) synaptic neuropeptide release being dominated by kiss-and-run DCV exocytosis (Wong *et al.*, 2015; Bulgari *et al.*, 2019; 2023; Klose *et al.*, 2021), and 2) the fate of postexocytosis DCVs differing between the traditional model of DCV transport and vesicle circulation: with the traditional model, postexocytosis DCVs would not undergo anterograde transport because that process is reserved for newly synthesized vesicles, while with circulation, both anterograde and retrograde transport are expected.

Therefore, DCVs were selectively labeled during kiss-and-run exocytosis by cell-specific expression of a neuropeptide tagged with a fluorogen-activating protein (Dilp2-FAP) and application of a membrane impermeant fluorogen (Bulgari *et al.*, 2019; 2023; Klose *et al.*, 2021; 2026). With this approach, fluorescence is only produced when the fluorogen diffuses through the fusion pore formed during kiss-and-run exocytosis to bind the FAP in the DCV lumen. At the neuromuscular junction (NMJ), where only a fraction of the DCV Dilp2-GFP content is released by each kiss-and-run exocytosis event (Wong *et al.*, 2015), there is colabeling of DCVs with Dilp2-GFP and Dilp2-FAP following kiss-and-run exocytosis (Bulgari *et al.*, 2019). Based on those prior experiments, fluorescent DCVs were imaged in distal sLNv axons following the ∼1-h incubation with the membrane impermeant fluorogen MG-TCarb while approaching the midmorning peak of synaptic DCV exocytosis and endogenous neuropeptide release at sLNv terminals (i.e., at ZT 2-3) (Klose *et al.*, 2021; 2026). In animals coexpressing Dilp2-FAP and Dilp2-GFP in sLNv neurons, FAP and GFP colocalized in moving axonal puncta (Figure 1B), thereby demonstrating postexocytosis labeling of DCVs in sLNv axons. Furthermore, imaging of multiple neurons in seven brain explants showed postexocytosis DCVs displayed both anterograde and retrograde axonal transport (Figure 1, B−D), which is consistent with delivery of neuropeptides to terminals by vesicle circulation (Supplemental Figure S1B).

**FIGURE 2:**
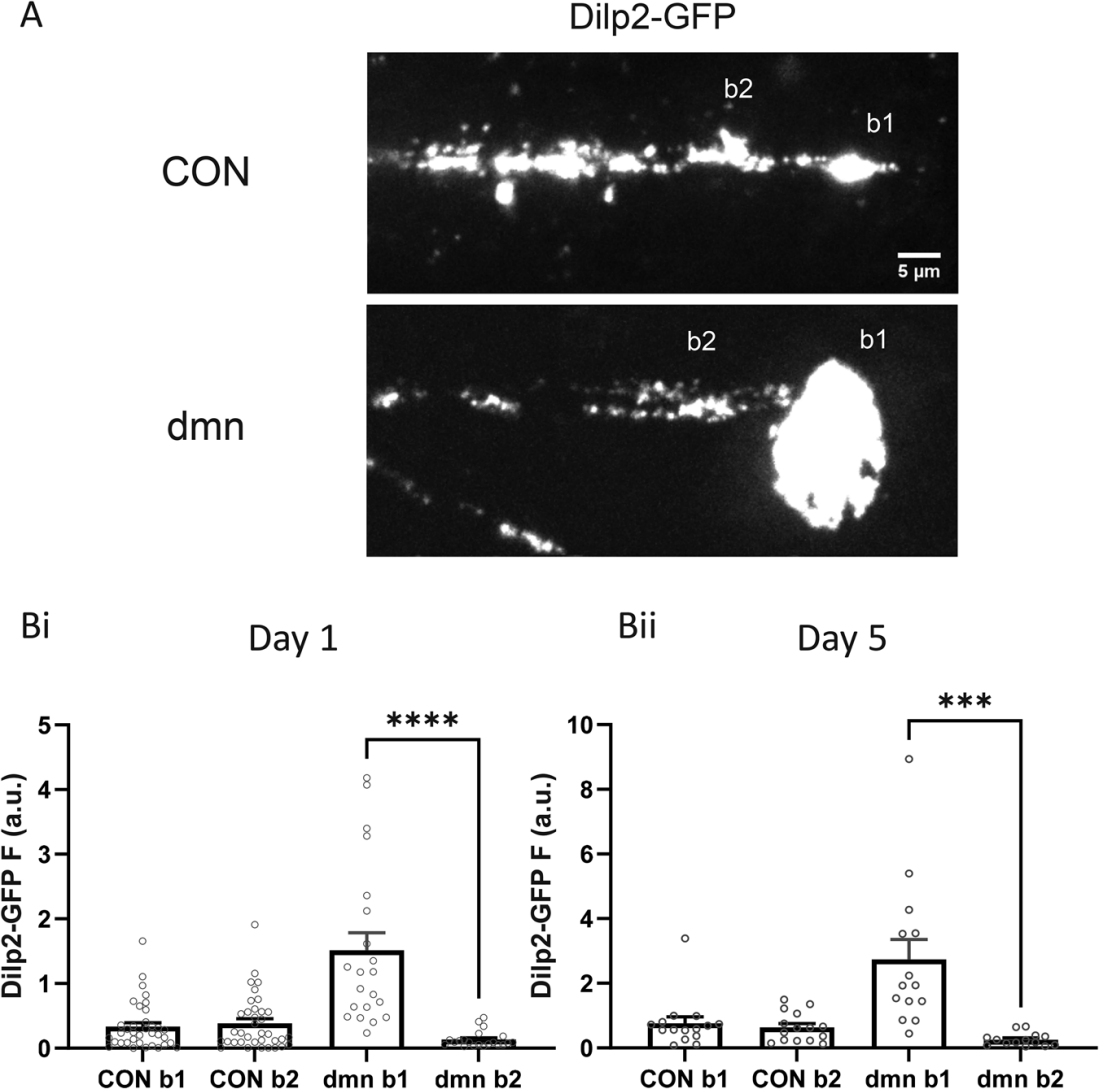
Dynamitin increases neuropeptide content of sLNv distal terminal boutons. (A) Images from distal sLNv nerve terminals of *UAS-Dilp2-GFP; PDF-GAL4* control flies (CON) and *UAS-dmn/UAS-Dilp2-GFP; PDF-GAL4* flies (dmn) at ∼ZT 2 on day 1 after eclosion. The most distal *en passant* bouton (b1) and its proximal neighbor bouton (b2) are labeled. Scale bar applies to both panels. (B) Dilp2-GFP fluorescence was compared between the most distal *en passant* bouton (b1) and its proximal neighbor bouton (b2) revealing a dramatic content difference between the two not seen in control terminals at the first (i) and fifth (ii) day posteclosion. ***P < 0.001, ****P < 0.0001, Paired *t* tests with Welch's correction for different variances. Points represent data from individual terminal branches (*n* = 36 CON and 20 dmn from 10 and 7 brain explants, respectively, in Bi, and *n* = 15 CON and 14 dmn from 6 and 5 brain explants, respectively, in Bii. Bar graphs in all figures show mean and SEM).

With vesicle circulation through *en passant* boutons, retrograde transport out of the most distal bouton removes excess DCVs supplied by anterograde transport (Wong *et al.*, 2012). Because dynein-mediated retrograde transport activation depends on the dynactin complex, this retrograde transport is inhibited by overexpressing the dynactin 2, p50 subunit (also known as dynamitin, dmn) (Burkhardt *et al.*, 1997), resulting in the accumulation of neuropeptide-containing DCVs in the most distal *en passant* bouton (Wong *et al.*, 2012). Therefore, to independently test whether DCVs undergo vesicle circulation in sLNv terminals, the approach of Wong *et al.* (2012) of using *UAS-dmn* to overexpress the dynactin subunit was applied to *PDF > Dilp2-GFP* animals. In the most distal sLNv boutons (indicated as b1 in Figure 2A) in *PDF > Dilp2-GFP* animals (Figure 2A; CON) examined during either the first day or the fifth day following eclosion, neuropeptide content is not different than their proximal neighboring boutons (CON b1 vs. CON b2; Figure 2, A and B [i and ii]). However, dmn overexpression to inhibit retrograde transport resulted in ∼10-fold more neuropeptide accumulation in the most distal bouton at both time points (dmn b1 vs. dmn b2; Figure 2, A and B [i and ii]). Thus, a genetic perturbation establishes that DCVs in sLNv terminals are delivered to boutons by vesicle circulation.

### Rhythmic capture of circulating DCVs in sLNv terminals

**FIGURE 3:**
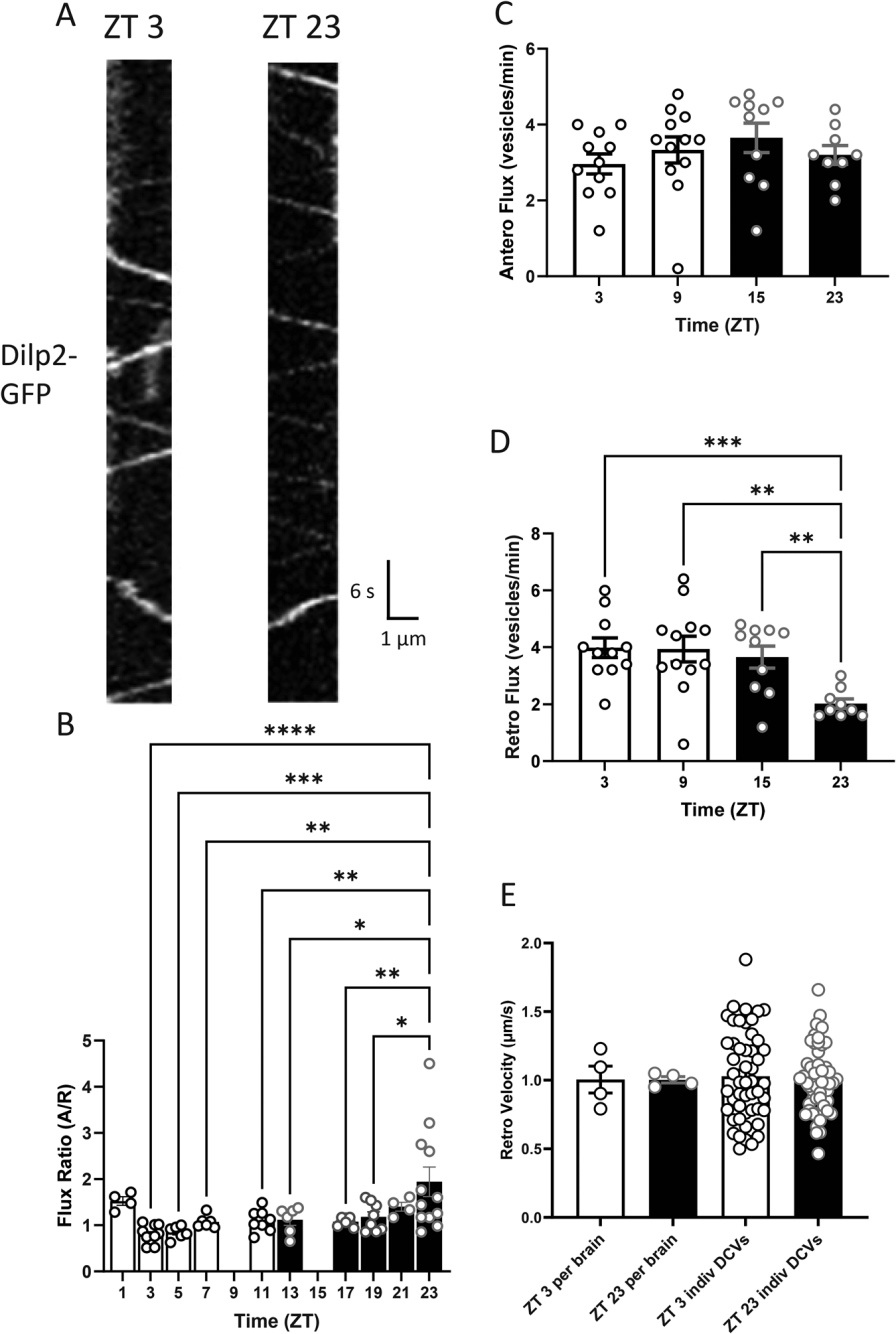
DCV flux and velocity show late-night capture upregulates synaptic neuropeptide content in sLNv nerve terminals. (A) Kymographs reveal both anterograde (slope down, left to right) and retrograde (slope down, right to left) DCV axonal transport in sLNv projections of *PDF > Dilp2-GFP* flies at ZT 3 and ZT 23. (B) Flux ratios (anterograde/retrograde) at different times of the day. *N* values (number of brain explants) for ZT 1, 3, 5, 7, 11, 13, 17, 19, 21, and 23 are 4, 10, 6, 6, 8, 6, 8, 8, 4, and 12, respectively. One-way ANOVA revealed a significant difference (*P* < 0.001). Posttest analysis by Dunnett's multiple-comparison test is presented: ****P < 0.0001, ***P < 0.001, **P < 0.01, *P < 0.05. (C) Anterograde flux and (D) Retrograde flux (vesicles/min) in *PDF > Dilp2-GFP* brains. For C and D, the *N* values (number of brain explants) for ZT 2-4, 8-10, 14-16, and 23 are 11, 12, 10, and 9, respectively. *P* < 0.01, Brown−Forsythe ANOVA. ***P < 0.001, **P < 0.01, Dunnett's T3 multiple comparisons test. (E) Retrograde velocity of DCVs at ZT 3 and 23 in *PDF > Dilp2-GFP* brains (left, average per brain; ZT 3 *N* = 4, ZT 23 *N* = 4; right, individual vesicle measurements: ZT 3 *n* = 49, ZT 23 *n* = 51).

To test for regulated axonal transport of DCVs, we initially measured the number of DCVs labeled with Dilp2-GFP being transported per minute (i.e., flux) and calculated the ratio of anterograde to retrograde DCV flux (A/R). Kymograph analysis showed that the flux ratio changed (*P* < 0.001, one-way ANOVA) with a peak at ZT 23 (Figure 3, A and B). Therefore, there is a net increase in axonal transport of DCVs to sLNv terminals late at night in preparation for the midmorning burst of synaptic neuropeptide release.

Anterograde and retrograde DCV transport fluxes were then examined in independent experiments to determine which component of the flux ratio changed, the numerator (A) or the denominator (R). Analysis of axonal transport showed that anterograde DCV flux (A) was constant at all times tested (Figure 3C), thus ruling out rhythmic changes in the ratio being driven by changes in anterograde axonal transport from the soma. Rather, retrograde axonal DCV flux (R) cycled (*P* < 0.01, one-way ANOVA) with a minimum late at night at ZT 23 (Figure 3D). This drop in retrograde flux is not explained by exocytosis-induced DCV depletion since the flux drop precedes daily synaptic DCV exocytosis and native synaptic neuropeptide release by several hours (Klose *et al.*, 2021; 2026). Furthermore, the decrease in retrograde flux occurred without altering the velocity of DCVs undergoing retrograde axonal transport (Figure 3E), thereby excluding a slowing of the retrograde transport motors. The observed reduction in retrograde flux hours before synaptic release with no change in retrograde velocity is indicative of increased synaptic capture of circulating DCVs (Shakiryanova *et al.*, 2006; Wong *et al.*, 2012; Cavolo *et al.*, 2016). Therefore, increased DCV capture, as opposed to greater DCV delivery by anterograde transport, produces the daily increase in synaptic neuropeptide content.

### DCV capture is independent of rhythmic activity, IP_3_ signaling, and axon plasticity

**FIGURE 4:**
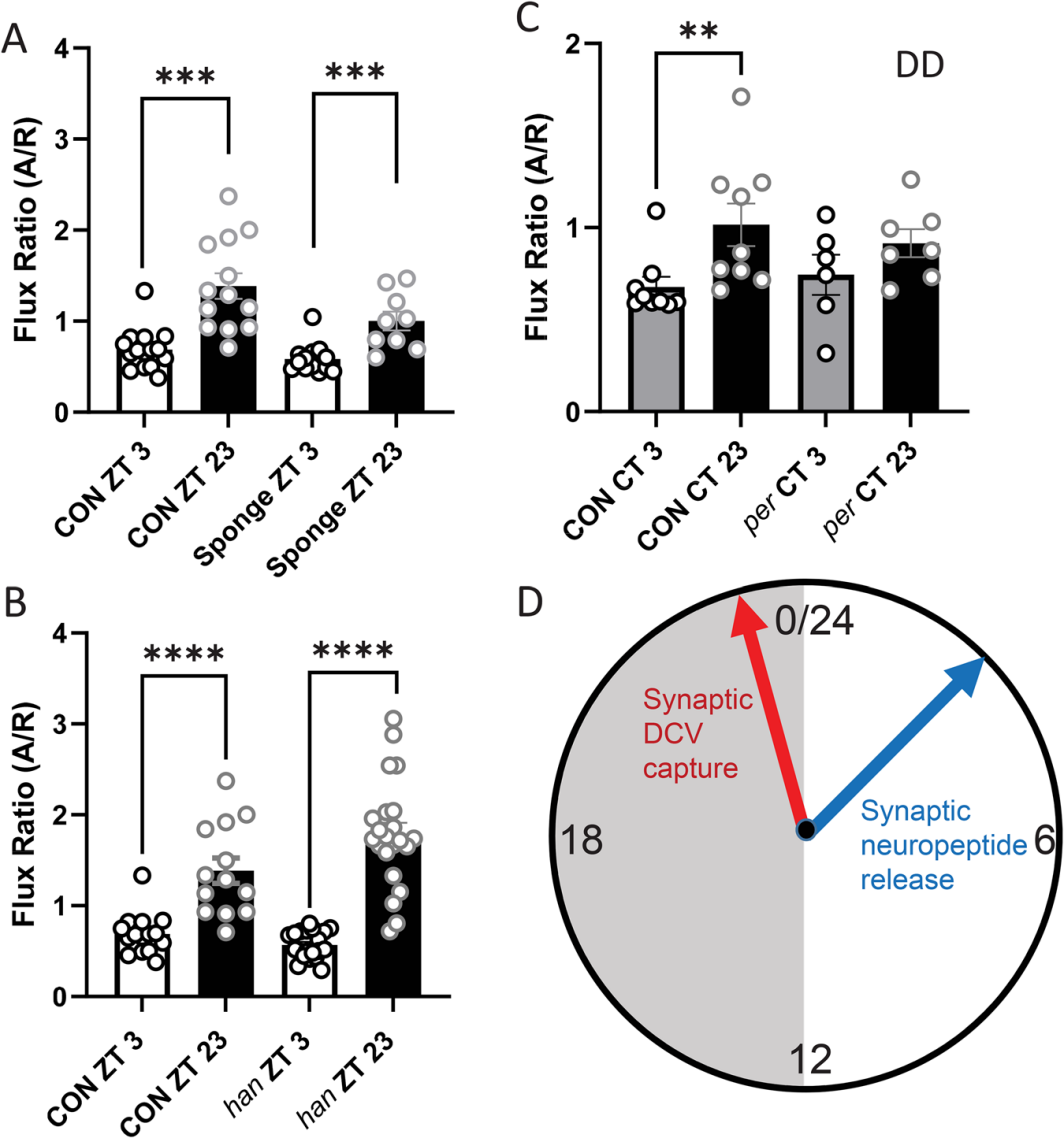
Rhythmic DCV capture requires the clock gene *per*, but not concurrent IP_3_ signaling or PDFR-dependent axonal plasticity. (A) Ratios of anterograde to retrograde DCV flux (A/R) at ZT 3 and ZT 23 in *w*; *UAS-Dilp2-GFP; PDF-GAL4* controls (CON) and IP_3_ sponge; *UAS-Dilp2-GFP; PDF-GAL4 UAS-IP_3_ sponge* sLNv axons (Sponge). *N* = 13 (ZT 3) and 9 (ZT 23) for CON; *N* = 15 (ZT 3) and 13 ZT (23) for IP_3_ sponge. For both CON and *han*. (B) Ratios of anterograde to retrograde DCV flux (A/R) at ZT 3 and ZT 23 in *w*; *UAS-Dilp2-GFP; PDF-GAL4* controls (CON) and *han*; *UAS-Dilp2-GFP; PDF-GAL4* PDFR null (*han*) sLNv axons. *N* = 15 (ZT3) and 13 (ZT 23) for CON; *N* = 20 (ZT 3) and 22 (ZT 23) for *han*. (C) Ratios of anterograde to retrograde DCV flux (A/R) 2 d after transition to constant darkness (DD) at CT3 and CT23 in *w*; *UAS-Dilp2-GFP; PDF-GAL4* controls (CON) and y^1^per^01^w*; *UAS-Dilp2-GFP; PDF-GAL4* (*per*) sLNv axons. *N* = 9 (CT 3) and 9 (CT 23) for CON; *N* = 6 (CT3) and *N* = 7 for (CT 23) in *per* animals. All data in this figure were analyzed using the Mann−Whitney test. **P < 0.01, ***P < 0.001, ****P < 0.0001. (D) Timing of presynaptic events in sLNv terminals. Arrows indicate peaks of clock-dependent synaptic DCV capture demonstrated here and synaptic DCV exocytosis and activity-dependent native neuropeptide release demonstrated previously (Klose *et al.*, 2021; Klose et al, 2026).

The late-night (ZT 23) synaptic capture of DCVs precedes the daily rise in Ca^2+^ spiking, which peaks 4 h later at midmorning (Klose *et al.*, 2026). However, Ca^2+^ release from endoplasmic reticulum participates in activity-dependent DCV capture at the NMJ (Wong *et al.*, 2009), and there is IP_3_-IP3R signaling in sLNv neurons at ZT 23 (Klose *et al.*, 2021). Therefore, we tested for the role of IP_3_ signaling in late-night synaptic DCV capture. Specifically, the flux ratio at ZT 23 was determined after cell-specific expression of the IP_3_ sponge (Uchiyama *et al.*, 2002), which inhibits IP_3_ signaling in sLNv neurons (Klose *et al.*, 2021). However, the IP_3_ sponge did not block capture at ZT 23 (Figure 4A). Therefore, Ca^2+^ elevation induced by either activity or IP_3_ signaling is not required for late-night DCV capture in the presynapse.

We then considered whether the rhythmic DCV capture is linked to daily morphological plasticity in the sLNv axonal arbor (Fernández *et al.*, 2008), which is associated with varicosities containing more DCVs (Ispizua et al., 2025). For this purpose, DCV axonal transport was studied in a PDF receptor (PDFR) null mutant (*han*), which prevents daily changes in terminal morphology while preserving rhythmic changes in PDF (Herrero *et al.*, 2020). In PDFR null animals, the daily rhythmic changes in axonal flux ratio persisted (Figure 4B). Therefore, the daily rhythm in DCV capture occurs independently of PDFR expression and the accompanying axonal plasticity it drives.

### Rhythmic DCV capture requires the circadian clock

The PDFR null mutant also abolishes morning anticipation behavior (i.e., the increase in motor activity before sunrise) (Hyun *et al.*, 2005). Because rhythmic capture occurs concurrently with this clock-dependent increase in locomotor behavior but persists when morning anticipation is disrupted by the PDFR null mutation, we considered whether capture depends on the circadian clock. If capture is independent of the circadian clock, rhythmic capture would likely be driven through environmental cues, namely daily changes in light levels, and thus would be disrupted after shifting to a constant level of illumination. In contrast, if daily capture depends on the circadian clock, it should persist after a shift to constant illumination. In fact, after shifting to constant darkness (DD), capture remained different between CT 23 and CT 3 (Figure 4C, CON), which is suggestive of clock dependence. To further test for the role of the circadian clock under DD conditions, we examined the effect of a null mutation in the *period* gene (*per^01^*), which is a critical component of the molecular clock. Consistent with the loss of oscillation in synaptic PDF content with this mutant (Park *et al.*, 2000), capture under DD conditions did not significantly increase in *per^01^* animals at CT 23 (Figure 4C, *per*). Therefore, the circadian clock controls the rhythmic increase in the presynaptic neuropeptide pool by vesicle capture hours in advance of midmorning activity-dependent synaptic neuropeptide release (Figure 4D; Klose *et al.*, 2021; 2026).

### Conclusions

In *Drosophila* sLNv clock neurons, there is a daily posttranscriptional surge in synaptic PDF content (Park *et al.*, 2000). Because native neuropeptide transport cannot be measured dynamically, we imaged DCV-specific reporters (Dilp2-GFP and Dilp2-FAP) that recapitulate native neuropeptide accumulation and release at sLNv terminals (Klose *et al.*, 2021; 2026). Furthermore, previously established genetic perturbations were used to probe the mechanism of the daily surge in synaptic neuropeptide content. These experiments did not exclude developmental effects. However, some conclusions were independently verified: vesicle circulation was supported by the observation of bidirectional transport after exocytosis (Figure 1) as well as by the distal effect of inhibiting retrograde transport (Figure 2), and the clock's involvement was consistent with data obtained in constant darkness as well as by the effect of the *per* mutant (Figure 4C). Furthermore, the lack of an effect of the PDFR mutant on capture contrasts with its effects on morning anticipation and axonal plasticity, demonstrating that the latter is not required for daily capture. Taken together, the combination of imaging of DCV axonal transport in the brain with genetic manipulations demonstrates that clock-dependent capture of circulating DCVs elevates presynaptic neuropeptide content in sLNv neurons each day. Because synaptic neuropeptide release is proportional to presynaptic neuropeptide content (Bulgari *et al.*, 2014), midmorning peptidergic synaptic transmission that controls circadian behavior via PDF is promoted by clock-dependent late-night synaptic capture of circulating DCVs.

Clock-dependent vesicle capture likely also upregulates presynaptic sNPF, a neuropeptide that is copackaged in the same sLNv neuron DCVs as PDF and promotes nighttime sleep (Shang *et al.*, 2013; Yu *et al.*, 2025). Unlike PDF, sNPF gene expression in sLNv neurons is rhythmic (Park *et al.*, 2000; Kula-Eversole *et al.*, 2010; Ma *et al.*, 2021), suggesting that both neuropeptide synthesis in the soma and vesicle capture in terminals act synergistically to rhythmically upregulate presynaptic sNPF stores for peptidergic transmission. It will be of interest to determine how clock neurons in *Drosophila* and mammals utilize clock-regulated vesicle capture and changes in neuropeptide gene expression to control rhythmic behaviors.

Two mechanisms of DCV capture were discovered in the fly NMJ: constitutive capture, which maintains neuropeptide content during quiescence by sequestering DCVs undergoing bidirectional transport, and activity-dependent capture, which replenishes presynaptic neuropeptide stores for minutes following release by tapping into anterograde DCV flux (Shakiryanova *et al.*, 2006; Wong *et al.*, 2012; Cavolo *et al.*, 2016). In contrast with activity-dependent capture, capture in sLNv clock terminals is not induced by activity, but rather is driven by the clock hours in advance of future synaptic activity and release (Figure 4, C and D). This predictive DCV capture is independent of sLNv axonal plasticity (Figure 4B), thus excluding that daily neuropeptide accumulation simply reflects capture to occupy the expanding axonal arbor. In addition, daily DCV capture does not require IP_3_ signaling, which is concurrent with, but independent of, increased capture (Figure 4A). A future challenge will be to resolve how clock-induced vesicle capture operates compared with activity-dependent and constitutive capture mechanisms, which are not fully understood in *Drosophila*.

## MATERIALS AND METHODS

### Imaging

Flies were entrained for at least 72 h in a 12-h light:12-h dark (LD) schedule. Then, 4 to 9-day-old males were dissected to generate *ex vivo* brain explant preparations. Dissections during the dark phase were performed under a red light. Adult flies were anesthetized with CO_2_ gas and brains were dissected in 0 mM Ca^2+^ HL3 saline solution (70 mM NaCl, 5 mM KCl, 20 mM MgCl_2_*6H_2_O, 115 mM Sucrose, 5 mM Trehalose, 5 mM HEPES, and 10 mM NaHCO_3_, pH 7.3) and *ex vivo* brain explants were then put into polylysine-coated plastic dishes containing HL3 with 2 mM Ca^2+^ (Klose *et al.*, 2016). sLNv somas are located on the ventral side of the *Drosophila* brain and send axons to the dorsal protocerebrum (Helfrich-Förster *et al.*, 2007). Therefore, brain explants were positioned so that dorsal sLNv terminals were viewed from the posterior surface of the brain, thereby enabling the imaging of DCVs in the distal axon with the dipping water immersion objective of an upright spinning-disk confocal microscope. Imaging was done on setups with an upright Olympus microscope equipped with a 60 × 1.1 NA dipping water immersion objective, a Yokogawa spinning-disk confocal head, a Teledyne Photometrics sCMOS camera, and lasers for illumination (488 nm for GFP and 640 nm for FAP). In some experiments, a recombinant with UAS-Dilp2-GFP and UAS-Dilp2-FAP was used so that GFP could be viewed for focusing before application of the membrane impermeant fluorogen MG-TCarb (Klose *et al.*, 2021). For DCV axonal transport, two axonal regions in the dorsal protocerebrum were imaged in at least one brain explant hemisphere. When possible, data from both hemispheres were collected and merged. Thus, an *N* of one represents a single brain explant. Time-lapse experiments entailed acquiring 180 images with 35 to 150 ms exposure times at 3 Hz. From these data, at least two regions of interest (ROI) where dorsally projecting axons defasciculated from bundles were used for generating kymographs with the Kymograph plugin of ImageJ. Kymographs were then used to quantify flux and, in some cases, DCV velocity. Unlike DCV diameter, DCV velocity, which is evident from the slopes in kymographs, is not limited by the diffraction limit. Quantification was done in ImageJ or Fiji. Statistical analysis (i.e., tests and calculations of mean and SEM for error bars) was performed with GraphPad Prism software.

### Fly lines

All flies used the *PDF-GAL4* promoter on the third chromosome (provided by Paul Taghert, Washington University in St. Louis). *UAS-Dilp2-GFP* and *UAS-Dilp2-FAP* were reported previously (Wong *et al.*, 2012; Bulgari *et al.*, 2019). *w^1118^* flies were from Zachary Freyberg (University of Pittsburgh). Fly lines from the Bloomington *Drosophila* stock center included *Bl# 8784* (*UAS-dmn* or *UAS-DCTN2-p50.D*), *Bl#* 33068 (*han*), and *Bl#* 80917 (*per^01^*). The IP_3_ sponge line was provided by C. Andrew Frank (University of Iowa).

## Supporting information

Supporting Video 1 Movie S1 Time-lapse experiment showing bidirectional axonal transport of sLNv DCVs. 110 images were acquired at 3 Hz. Downward transport is in the anterograde direction. Upward transport is in the retrograde direction.

## Abbreviations used:

CT: circadian time
DCV: dense-core vesicle
DD: environment maintenance in continuous darkness
NMJ: neuromuscular junction
sLNv neurons: PDF-containing small ventral lateral clock neurons
ZT: zeitgeber time.
