# Supplementary material for: Anticipatory capture of circulating peptidergic vesicles in a clock neuron
Source: Mol Biol Cell. 2026 Apr 6;37(5):br12. doi: 10.1091/mbc.E25-11-0558 (PMC13202839; doi:10.1091/mbc.E25-11-0558)
Supplement: Supplementary file 2 [file mbc-37-br12-s001.pdf]

Supplemental Materials

Molecular Biology of the Cell

Klose *et al*.

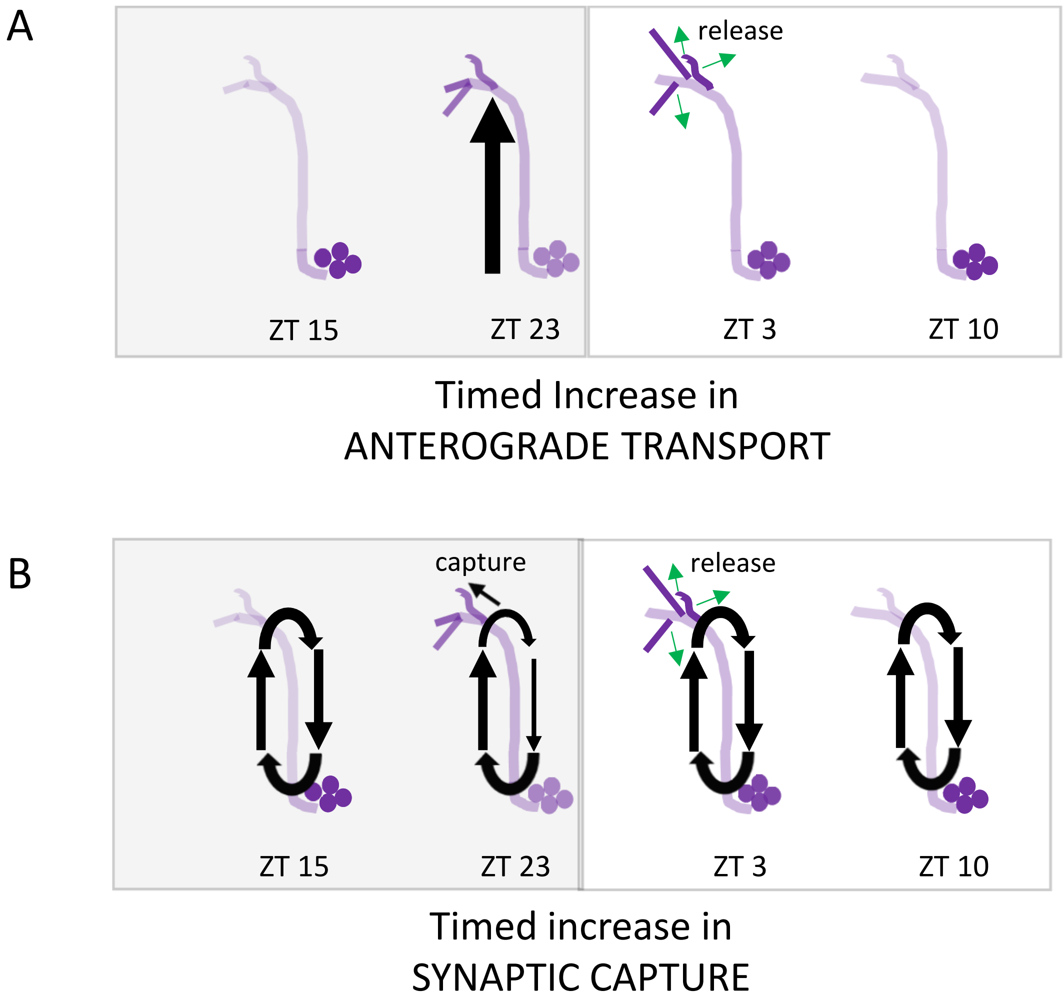

Figure S1. DCV transport in sLNv nerve terminal projections. Schematics present two hypotheses for how DCV transport dynamics could increase neuropeptide content at sLNv nerve terminals late at night and prior to release. A. Timed increase in anterograde transport of DCVs to nerve terminals late at night. B. Timed increase in synaptic capture of circulating DCVs late at night resulting in reduced retrograde transport.
